# Home-grown school feeding: assessment of a pilot program in Nepal

**DOI:** 10.1186/s12889-019-8143-9

**Published:** 2020-01-08

**Authors:** Rachana Manandhar Shrestha, Pepijn Schreinemachers, Mamta Gurung Nyangmi, Manoj Sah, Judy Phuong, Shraddha Manandhar, Ray-Yu Yang

**Affiliations:** 1 Present Address: Department of Community and Global Health, University of Tokyo, Tokyo, Japan; 2 Present Address: Independent Consultant, Kathmandu, Nepal; 3 Enabling Impact Program, World Vegetable Center, Bangkok, Thailand; 4 Education Support, World Food Programme, Lalitpur, Nepal; 5 Present Address: Global Survey: Asia and Oceania, Global Child Nutrition Foundation, Seattle, Washington USA; 6 Present Address: Helen Keller International, Kathmandu, Nepal; 7 Healthy Diets Program, World Vegetable Center, 60 Yi-Min Liao, Shanhua, Tainan, 74151 Taiwan, Republic of China

**Keywords:** School meal, Dietary quality, Nutrition and health, Children, Agriculture, Impact, Nutrition-sensitive learning

## Abstract

**Background:**

The Nepal School Meals Program reached 600,000 schoolchildren in basic education in 2017 and plays a key role in the government’s strategy to increase children’s academic and nutritional outcomes. A large part of the program is implemented through cash transfers with schools responsible for the school meal delivery. Home-grown school feeding, an approach in which local communities are given greater control over the school meals program and part of the food is sourced locally, may strengthen local ownership and improve meal quality, but there is a lack of evidence for impact.

**Methods:**

This study piloted home-grown school feeding in 30 schools reaching nearly 4000 children in Sindhupalchok and Bardiya districts in Nepal with the aim to assess operations and outcomes in comparison to the regular cash-based school meals program. The study used a one-time post evaluation with a mixed methods approach. Qualitative data were collected through 12 focus group discussions and 28 key informant interviews with government and school staff, parents, cooks, cooperative members, World Food Programme representatives and other stakeholders involved in the pilot program. The quantitative part applied a quasi-experimental design and used cross-sectional data collected from 1512 children in 30 pilot and 30 control schools.

**Results:**

The quantitative data indicated that children in the pilot schools had a significantly higher provision of midday school meals (+ 19%; *p* < 0.01) and a higher school meal quality in terms of dietary diversity (+ 44%; *p* < 0.01) and nutritional content (e.g. a 21%-points higher consumption of vitamin A-rich fruit and vegetables; *p* < 0.01). The qualitative data identified key drivers of these positive outcomes as the use of standard meal options, capacity building of local stakeholders, strengthened community ownership and accountability mechanisms, and local food supply chains. Maintaining the observed gains would require a 20–33% increase in the current budget per school meal in addition to the cost of capacity building.

**Conclusions:**

This study for Nepal shows that home-grown school feeding strengthened operations of the school meals program and led to a significantly higher meal provision and quality of school meals.

## Background

Child malnutrition is a major cause of morbidity and mortality among children in Nepal and impedes their long-term physical and mental development [1]. An estimated 32% of adolescents are stunted [2]. The country has about 5.2 million children (5–12 years old) in primary or lower secondary school, but only 87% of the children who start primary school go on to grade 5 and only 75% go on to grade 8 [3, 4].

School meals programs are widely considered as one of the most effective interventions to simultaneously improve nutrition and education outcomes for schoolchildren in developing countries [5]. School meals programs contribute to alleviating short-term hunger in schoolchildren, which increases their ability to concentrate and learn while they are at school [6, 7]. Such programs also increase student enrolment, attendance and retention rates [8]. Jointly, these factors contribute to better academic outcomes as well as improved child health [9].

School meals programs are commonly implemented either through direct food provision or through cash transfers to schools and this study focuses on the latter. Cash-based school meals give more autonomy to schools, but sometimes face challenges such as varying meal quality, misuse of funds, delays in the cash flows disrupting meal provision, and may distract teachers from classroom teaching. To address these issues and make the program more sustainable, there is an increasing interest to give communities greater control over the program implementation. Part of this strategy involves purchasing locally produced food to support communities and strengthen local food supply systems. This approach has become known as home-grown school feeding (HGSF) [8, 10].

In Nepal, the school meals program (SMP) is a key strategy of the government to abate malnutrition, as formulated in the School Sector Development Plan (2016–2022) and the National School Health and Nutrition Strategy [11]. It has been shown that every dollar invested in the program generates economic value of US$ 4.1–5.2 over the lifetime of a beneficiary child [12].

The Nepal SMP serves a midday meal to over 600,000 children according to data for 2017 [13]. In highly food insecure districts it is implemented as food transfers and provides children from pre-primary to grade 8 a ration of nutritionally-enhanced food for 200 days a year. In other districts it is implemented as a cash transfer program, which supplies meals for 180 days a year. Schools located in the hills and plains receive Nepalese rupees (NPR) 15 per child per meal (~ 0.13 US$) and schools in the mountains receive NPR 20 (~ 0.18 US$) to serve a midday meal for children from pre-primary to grade 5 [14]. The cash-based SMP in Nepal faces similar challenges as mentioned above and the government initiated a pilot SMP in 2017 with alternative design toward a home-grown school feeding.

There is an urgent need for better evidence for home-grown school feeding in Nepal. This study therefore reports on a pilot SMP in which stakeholders contributed to a better design with greater involvement of parents and local farmers and aimed at improving the nutritional quality of midday school meals. The objective of this study is to compare this alternative SMP design against the regular cash-based SMP in terms of program outcomes and effectiveness. We do this using a mixed-methods approach, combining quantitative and qualitative data, as a one-time ex-post evaluation of outcomes and operations.

## Methods

### Intervention

The pilot being tested aimed to improve the current cash-based SMP. The pilot was implemented by the Centre of Education and Human Resource Development with technical support from the World Food Programme (WFP). SMP stakeholders identified challenges to the current program, such as the lack of funds, occasional mismanagement of funds, poorly trained cooks and program managers, and lack of knowledge about and access to nutritious food. Intensive consultation between national and sub-national stakeholders identified five areas of improvements to the school meal provision, which were tested as a part of the pilot [15]:

1. The school was connected to a local agricultural cooperative to promote the use of locally produced food, enhance smallholder farmers’ access to a reliable market and strengthen the monitoring of resource use.
2. Parents, caterers, cooks and teachers were trained in commodity management, nutrition and food preparation as well as basic accounting and record keeping, kitchen gardening, WASH (water, sanitation and hygiene) and monitoring. Thirteen standard meal options with recipes were developed that meet the nutrition standards for a mid-day school meal as defined by the government of Nepal. The recipes were diverse and nutritionally balanced and within the means of the budget. Each school selected six meal, one for each school day.
3. Basic kitchen infrastructure was improved with a standard set of kitchen utensils and energy efficient stoves.
4. Resources were allocated to hire a professional food preparer.
5. Children in grades 1–3 received complementary education for 4 months in nutrition-sensitive literacy (NSL) promoting healthy food and hygiene behavior. This included specially designed learning materials and teacher trainings in how to use these materials.

The program implementers realized that schools differ in their capacity to manage the SMP. To cater for this, three variations, or modalities, were tested:

1. *Parents association modality:* The parents association manages the program with parents taking turns to cook and distribute meals on site using a roster system. The school management committee provides oversight.
2. *Catering modality*: Meals are prepared by one or more caterers who are employed by the school management committee. In smaller schools, the caterer may cook at home and manages everything from food procurement to meal distribution, while in larger schools the cooking is usually done in the school. The school management committee provides oversight.
3. *School-managed modality*: The school management committee itself manages the program, preparing and distributing food on site, which is similar to how the regular cash-based program is typically implemented, but with additional support from local agricultural cooperatives.

The pilot was implemented in 30 schools in Bardiya and Sindhupalchok districts, which were already part of the government’s cash-based SMP [14]. Pilot schools were not selected randomly, but were selected based on the presence of agricultural cooperatives, an active school management committee, adequate infrastructure, and were clustered in no more than four local administrative units per district for ease of access. All 30 schools implemented the five common areas of improvements and one modality for managing the SMP. Larger schools generally have better capacity and facilities for managing and implementing the program. They tended to choose either the catering modality or the school-managed modality. Smaller schools tended to opt for the parents association modality as they required parents’ involvement and assistance in implementing the program. The pilot ran for 13 months and was targeted at primary schoolchildren in grades 1–5 (aged 5 to 9 years old).

### Assessment

A convergent mixed-methods approach, combining qualitative and quantitative data, was used to conduct a one-time ex-post evaluation of outcomes and operations. Data were collected from September to November 2017.

#### Qualitative method

The qualitative approach aimed to gain an in-depth understanding of how the intervention was implemented, what changes it brought about, and the challenges that were encountered. Qualitative data were only collected from pilot schools, and included focus group discussions (FGDs) and key informant interviews (KIIs).

The objective of FGDs was to understand participants’ perspective on the pilot [16]. FGDs comprised of 5–12 participants per session and included different stakeholders from the school and community levels. The average time taken for FGD sessions was around 1.5 h. KIIs were conducted with the stakeholders at national, districts as well as schools and community levels with the participants who have particularly informed perspectives on our study topic [17]. KIIs helped to collect detailed information about each stakeholder’s perspective on the pilot SMP implementation. KII sessions lasted for an average of 1 h.

Separate question guides were developed for the FGD, for the KII at the national and district level, and for the KII at the school and community level (for detailed guides, please see Additional file 1). These guides were based on the ‘Global School Feeding Sourcebook: Lessons from 14 countries’ [18] as well as the analytical framework developed in the study in consultation with SMP stakeholders. The guides were written in English, translated into Nepali and back-translated into English by different translators. Appendix 3 shows the key thematic areas of the guides.

The first author, who had prior experience with qualitative data collection, conducted all FGDs and KIIs. FGDs and KIIs were repeated until data saturation was reached and new information stopped emerging. A total of 12 FGDs and 28 KIIs were conducted (see Appendix 1 and 2).

#### Qualitative data analysis

The first author discussed with co-authors the readability and understandability of the question guides after the first two data collection rounds. The first author added probing questions during data collection when necessary. Notes were taken and FGD and KII sessions were audio-recorded. Notes were reviewed and verified with participants at the end of each session. Interviews were conducted until data saturation was reached [18]. The consolidated criteria for reporting qualitative research guideline was used to report the data [19]. Data was first transcribed into Nepali. Drawing on principles of the framework approach to qualitative data analysis, coding and analysis was conducted [20]. NVivo (version 11) was used for data coding. The first author and a co-author double-coded four transcripts (10%) and had iterative discussions to come up with a preliminary common coding framework. The researchers then divided the 40 transcripts into two equal sets for each coder. New codes were added after discussion and a final coding framework was developed [21]. Themes, codes and sub-codes from the data were identified and analyzed for data patterns [21]. Please see the codebook in Additional file 2. 

#### Quantitative method

The quantitative approach assessed the effect of the pilot on nutritional and educational outcomes. The analysis compares the group of pilot schools against a group of control schools. It did not compare between the modalities within the pilot because the number of schools per modality is not enough to do such comparison. The analysis uses a quasi-experimental design to attribute outcomes to the intervention. The study applied the same criteria to select control schools as had been used to select pilot schools to ensure that the groups were comparable. The drawback is, however, that the results of our study cannot be generalized to all schools in the cash-based SMP.

The pilot intervention reached 3935 schoolchildren. The size of schools in the pilot ranged from 18 to 412 children per school with an average size of 123 children (SD 98). We used Power and Precision software (version 4; Biostat, Englewood, NJ, USA) to calculate the required sample of children. This was estimated to be around 635 for the pilot and for the control schools. To account for missing data, we aimed for a sample of about 700 children per group.

Data were collected from children in the age range of 5–12 years old from grades 1–5 in the 30 pilot schools and a comparison group of 30 schools outside the pilot. Data were collected from 20 children per school in Sindhupalchok District and from 40 children per school in Bardiya District where schools are generally larger. Stratified random sampling was used with grade and sex used as strata.

Face-to-face interviews were done with children in grades 1–3. Each child was interviewed individually by a trained enumerator and this took about 30 min per interview. Children in grades 4–5 completed a self-administered questionnaire in Nepali in school during school time. The enumerators provided instructions to the children and remained present to answer questions and to prevent children from working together.

#### Outcome variables and questionnaires

The outcome variables are described as follows:

1. *Hygiene practice score:* Measured using 5 multiple choice questions for grades 1–3 and 7 multiple choice questions for grades 4–5. Responses were coded as correct (1) or incorrect/don’t know (0) and summed.
2. *Nutrition and hygiene knowledge score*: Measured using 10 multiple choice questions about nutrition and hygiene using photos. Responses were coded as correct (1) or incorrect/don’t know (0) and the score ranged from 0 to 10. Different tests were developed for grades 1–3 and grades 4–5.
3. *School meals received:* Measured by asking children the number of school meals they had received during the last six school days as the cash-based school meal program is implemented for 6 out of 7 days of the week (from Sunday to Friday)
4. *Fruit and vegetable knowledge score*: Measured using 10 photos of common fruit and vegetables with children asked to write down the name of each. Responses were coded as correct (1) or incorrect/don’t know (0) and summed over all questions. The score ranged from 0 to 10.
5. *Dietary practice score*: Children in grades 4–5 were asked to indicate how frequently (never, rarely, sometimes, everyday) they consumed food from five types of foods (milk/curd, meat/fish, egg, fruit, junk food). The responses ranged from ‘1 = never’ to ‘4 = everyday’ and summed over all questions. The score ranged from 4 to 20. This was simplified for children in grades 1–3 by showing them photos of eight food groups and asking them to tick off the food groups they ate almost every day. The responses were coded as ‘1 = yes’ and ‘0 = no’ and summed up. The score ranged from 0 to 8. In both cases, a higher score indicates better dietary practices.
6. *Knowledge of healthier snacks:* Children in grades 1–3 were asked five pictorial questions, each showing two alternative snack choices—one healthier one (coded as 1) and one less healthy one (codes as 0). The score ranged from 0 to 5.
7. *Dietary diversity:* The dietary diversity of children’s food consumption on the previous day was measured using a retrospective one-day food record method. Children recorded the number of meals (including snacks between meals) and the types of food they had consumed over the day before the survey. Responses were categorized into seven food groups: (a) grains, roots and tubers, (b) legumes and nuts, (c) dairy products, (d) eggs, (e) vitamin A rich fruits and vegetables, (f) other fruits and vegetables, and (g) flesh foods. If a child had consumed the food belonging to the particular food group, a score of ‘1’ was given for that food group and ‘0’ score was given if not consumed. Therefore, the sum of food groups was calculated, ranging from 0 to 7 with a higher score meaning greater dietary diversity. Dietary diversity of the school meals was calculated in the same way.

The survey also recorded children’s gender, age, grade, language spoken at home and living arrangements. Student-level questionnaires were developed in consultation with program implementers and school teachers. The questionnaires were first developed in English by adapting already tested questionnaires, including the Global School-based Health Survey questionnaire [22], the ‘wash in schools: monitoring package’ [23], a student questionnaire previously used for process monitoring by the WFP, a dietary diversity questionnaire [24], and a questionnaire previously used for evaluating a school garden project in Nepal [25].

Separate questionnaires were developed for children in grades 1–3 and those in grades 4–5 as the younger children were found to have low literacy skills and thus required face-to-face interviews while the older children were able to complete a self-administered questionnaire. Questionnaires were translated into Nepali and back-translated into English by a different translator to ensure that the translation was correct. The questionnaires were pre-tested in two schools in Sindhupalchok District and modified, also using feedback from public health experts and school teachers.

#### Quantitative data analysis

Quantitative data were collected using paper-based questionnaires, entered in Epidata (version 3.1), and analyzed using SPSS (version 16.0). The presence of selection bias was tested by conducting chi-square tests on children’s socio-demographic variables (age, grade, gender, living arrangements, language spoken at home). None of the coefficients were significant (*p* > 0.05), which suggests that selection bias in observable characteristics is not a problem in the data. Therefore, Chi-squared and unpaired two-tailed t-tests were used test for significant difference in mean outcomes variables between children in the pilot and the control. The quantitative method did not compare between modalities because the sample of schools per modality was not large enough.

#### Assessment of pilot school meal costs

The cost of school meals included the cost of food, fuel, transportation and the wage of a cook. The cost of training cooks and teachers was not included. Cost analysis of the pilot was based on an internal project report of the Education Support Unit (WFP Nepal, March 2018), which showed the expenditures on school meals as based on the schools’ monthly financial reports. The food expenditures as reported by the schools were verified with data from the cooperatives that supplied the food. Data on the cost of the cook cost came from the cooperatives, which paid the cook.

The cost per meal per child was calculated by dividing the total meal cost by the number of actual feeding days and the average number of students present on these days. These data were provided by the schools and verified with the cooperatives. The pilot did not start in the same month in the three locations and the average meal cost therefore was calculated over a 12-month period in Mahankal, an 8-month period in Thulosiruwari, and a 5-month period in Bardiya.

#### Assessment of school meal quality

The nutritional content of the school meals was assessed for the four most frequently chosen standard meal options using standard food conversion tables of the ‘Global School Feeding Sourcebook: Lessons from 14 countries’ [18] and the Food Composition Table for Nepal (2012). The nutrient supply was compared to the recommended dietary allowance (RDA), which came from the Expert Committee of the Indian Council of Medical Research.

## Results

### Qualitative assessment of school meal program operations

Qualitative data were collected from 12 FGDs and 28 KIIs with SMP stakeholders at school/community, district and national levels. Conducting the FGDs with a heterogeneous group of school management, agricultural cooperative members, cooks and parents was challenging but manageable as we used a similar method in a previous study. The qualitative results are presented by the major themes that emerged from the data.

#### Menu development

Participants in the KIIs and FGDs mentioned that in the pilot SMP, the menus had been developed in a participatory fashion with local, district and national stakeholders with priority given to the use of nutritious and locally available food. This led to 13 standard meal options with specified ingredients and quantities from which each school selected six menus—one for each school day. The control SMP had no standard menus and is only guided by the government’s general mid-day meal program implementation procedure [26].

*“A standard menu was developed taking into consideration what local commodities could be easily procured. We worked with our nutritionists and we worked with local stakeholders to get that information. There is a nutrition tool. So, you can actually see with a certain quantity, what’s the nutritional need and based on that we tried to meet at least the basic need. It’s about a third of the nutritional content.”*

*-KII with national level participant, Kathmandu*

#### Food procurement, transportation and storage

Most participants from KIIs and FGDs mentioned that in the pilot SMP, the WFP contracted local cooperatives to assist the schools in food procurement and transportation, hired cooks were trained in nutrition and hygiene, supervised stock management and assisted in procurement. A short supply chain was established to procure local food from local kitchen gardeners or farmers. Food such as cooking oil, lentils, dry beans, and beaten rice were not produced locally and were procured from local retailers. This created stronger ties with local food producers and communities. Most cooks who participated in FGDs shared that the WFP-provided tin storage boxes were in use to store ingredients. The schoolteachers and members of the school management committee recognized the importance of these for food quality and safety.

*“The World Food programme has provided a tin box for storage. We safely store food in it and lock it. We also check the quality of the ingredients. If the quality is poor, we send the ingredients back.”*

*-KII with a cook, Bardiya*

#### Food preparation

Most of the participants in FGDs and KIIs agreed that trained people systematically prepared meals following the set menu. Pilot schools in the school-managed or parents association modalities had cooks who prepared meals on site, whereas in the caterer modality, the food was mostly prepared off-site and brought to the school by the caterer. Many participants mentioned that cooks had received training on what ingredients to use, how to estimate the correct quantities, how to prepare meals, cleanliness and hygiene practices, and food storage. Cooks tried to follow the menu, but some modifications were made based on seasonal food availability and cost without compromising nutrition content.

*“The foods that are prepared now are done so according to the menu. The cooks have also been given training for it. So, whatever ingredients the menu has asked us to use are all actually used when cooking. There is a uniformity in the lunch meals that are provided now.”*

*-FGD with school principals and focal teachers, Bardiya*

*“Most of the time we follow the menu. But in rare cases when a particular ingredient mentioned in the recipe is not available then we prepare the meal using the ingredients we have in stock.”*

*-FGD with school management committee members, parents and cooks, Bardiya*

#### Distribution to children

At the pilot schools, cooks and teachers distributed meals when these were prepared on-site, while parents or caterers distributed the meals when these were prepared off-site. Hygiene practices were incorporated and reinforced during meal distribution such as hand washing before and after eating. Full portion sizes, as defined in the standard menus, were distributed to children in grades 4–5, and smaller portion sizes (not defined, but based on judgement) were provided to children in grades 1–3.

*“We come and cook meals. We ensure sanitation while feeding children. We ask the children to wash hands first and then we give them food. We give them food only after they have washed their hands. We ourselves wash our hands with soap and water before preparing meals.”*

*-FGD with cooks, Sindhupalchok*

#### Community participation

The pilot SMP emphasized home-grown food production and community participation in the SMP program and the majority of participants in the FGDs and KIIs confirmed a high participation of parents, cooperatives, local farmers, retailers, cooks, schools, and school management committees.

*“There is a committee set up for the management of the School Meals Program. There is a school management committee, there are the teachers, parents and cooks. There is also the cooperative, which provides us with vegetables daily and the materials that are required for the menu. Schools on the other side, manage and monitor everything.”*

*-FGD with school principals and focal teachers, Sindhupalchok*

#### Capacity development

Through the FGDs and KIIs, participants at all levels mentioned that the WFP had provided local training to parents, caterers and cooks on commodity management, nutrition, food preparation, kitchen gardening, WASH and record keeping. Similarly, local cooperatives were also trained on accounting, billing and monitoring.

*“The trainings were very helpful. After the cooking training, I am very good at cooking and took less time.”*

*-FGD with SMC, parents and cooks, Bardiya*

*“I have taken the training on accounting, billing and monitoring, which was for 3 days. A few staff from the WFP had come to provide the training. In the past we didn’t know how to keep the records of our expenses.”*

*-FGD with cooperative members and cooks, Sindhupalchok*

#### Nutrition-sensitive literacy

Many school principals and focal teachers participating in the FGDs and KIIs were aware that the objectives of the nutrition-sensitive literacy (NSL) pilot were to create knowledge and advocate behavior change toward eating more nutritious meals in schools. They also mentioned that schools had received a Curriculum Development Center approved teachers’ guidebook and workbooks for children and that focal teachers had received training on how to use the NSL materials in the curriculum. According to the focal teachers, the NSL taught about food categories (food that protects, food that gives energy, food for growth). The school principals and focal teachers also mentioned that they organized NSL events such as cleanliness and sanitation rallies and a food and nutrition fair (called *mela*) where children and parents demonstrated nutritious and locally available food. The study found that teachers, parents and children had a positive opinion about the NSL.

*“NSL has a lot of benefits. Because the flashcards used, which is one of the teaching materials, it allows the children to learn easily. The guideline has a well-prepared work-plan which makes the work easier for us. Once we go through the guidelines and prepare 8-10 flash cards, we can run a class easily. And makes our work a lot easier.”*

*- KII with focal teacher, Sindhupalchok*

### Qualitative assessment of outcomes

The majority of school principals, focal teachers and parents who participated in the FGDs and KIIs were of the opinion that the introduction of the pilot increased children’s school attendance and retention rates. The frequency of children skipping afternoon class to go home for a midday meal decreased. It was furthermore observed that children developed improved dietary and hygiene practices such as a reduced consumption of junk food, increased hand washing at critical times, and better personal hygiene. Many teachers observed that children’s learning improved and they performed better as a result of the better-quality midday meal.

*“Children used to go home hungry at around 3 in the afternoon. The parents had a hard time feeding the children then. The program eased the rush children used to have to get home because of hunger. The children are also able to make time for study now. The biggest issue was the realization that students were hungry by the time they went home. Feeding children lunch has done wonders.”*

*- FGD with focal teachers, Bardiya*

*“Even though the children used to eat lunch, they had ready-made doughnuts and snacks available in the market. The nutritional value that they get is definitely better now that the cooperative is working continuously to provide us with the things that we require every other day. We have had a burden lifted from our shoulders, which has made it easier for us to work. I feel that it would have been difficult for us to manage those things if the World Food Programme and the cooperative had not supported us.”*

*-FGD with school principals & focal teachers, Sindhupalchok*

*“Earlier students used to be absent a lot. Nowadays students come to school regularly. Earlier children used to make a lot of mess in school. Then the World Food Programme came and focused on cleanliness and waste management. Then the school also started maintaining cleanliness in schools. Earlier, there were no water tanks in schools. But then there were talks that if we want children to form a habit of washing hands, water tanks need to be constructed.”*

*- FGD with Parents, Bardiya*

### Assessment of school meal cost

Both pilot and control schools received a budget of NPR 15 per child per meal for 180 school days. The budget allocation was on a quarterly basis and based on attendance records. In the pilot schools, teachers prepared monthly attendance reports, which were verified by the school principal, the school management committee, the agricultural cooperative, the resource person and the school inspector before it was handed over to the District Education Office. The schools checked the money spent by cooperatives on food and tallied it against the available budget of NPR 15 per child per meal. These findings show that the pilot schools had an increased oversight and better recording and verification mechanism. Before the pilot program, some schools would divert some of the school meals budget for other purposes.

The salary of cooks was paid by the WFP directly to the bank account of the agricultural cooperative. The allocated budget was managed with a focus on school meal quality, program implementation, and prevention of budget misuse. Also, in the pilot SMP, district level WFP staff ensured timely budget release for the pilot schools after verifying attendance reports.

The cost per child per meal in the pilot SMP ranged from NPR 21.0 for larger schools (over 150 children) to NPR 33.5 for smaller schools (less than 50 children) as shown in Table 1. Economies of scale in meal preparation relate to the hiring of the cook as this can be distributed over more children in larger schools. This also explains why the parent associations and the caterer-managed modalities are more expensive than the school managed modality. However, the parents association modality is critical for small schools where parents volunteer in turns to prepare meals to save money, which can then be used to purchase food.

**Table 1:** Meal costs borne by the school for the pilot school meal program, in NPR/child/meal, 2017

	Food	Fuel	Cook	Transportation	Total
By school size:					
- Less than 50 children	13.42	1.98	17.81	0.26	33.47
- 50 to 100	13.76	1.89	7.31	0.48	23.44
- 100 to 150	13.28	2.08	6.36	0.59	22.31
- More than 150	13.29	2.03	5.21	0.43	20.96
By modality:					
- Parents’ association	13.41	1.96	14.04	0.34	29.76
- Catering	13.61	1.99	9.46	0.18	25.24
- School managed	13.41	1.95	6.21	0.96	22.53

Data source: Internal report of the Education Support Unit (WFP Nepal; March 2018) and data collected from schools and cooperatives

### Assessment of school meal quality

Four popular menus were analyzed for their nutritional content using standard food conversation tables (Table 2). The menus included at least three food groups, namely a staple grain (rice, millet), a protein-source food (meat, egg, milk, beans/legumes) and vegetables (green leafy, high pro vitamin-A vegetables, and others). The midday meal supplied on average 19% of the daily energy requirement of girls at ages 10–12, 27% of their vitamin A requirement and 20% of their iron requirement.

**Table 2:** Average weight of school meals and their contribution to the recommended dietary allowance (RDA) of children 7–9 years and girls 10–12 years

Menu	Meal weight (grams)	% of RDA						
		Energy	Protein	Vit. A	Niacin	Thiamine	Calcium	Iron ^a^
Children 7–9 years:								
- Nutritious porridge	156	20	25	23	19	29	7	10
- Millet flour pancake	196	21	31	63	19	48	16	46 (14)
- Mix veg fried rice	117	18	22	28	16	19	8	9
- Rice flake with chicken curry	111	30	33	0	19	5	23	90 (23)
Mean	145	22	28	29	18	25	13	39 (13)
Schoolgirls 10–12 years:								
- Nutritious porridge	156	17	18	23	19	23	6	6
- Millet flour pancake	196	18	22	64	19	38	12	27 (8)
- Mix veg fried rice	117	15	16	20	16	15	6	5
- Rice flake with chicken curry	111	25	24	0	19	4	17	54 (14)
Mean	145	19	20	27	18	20	10	23 (8)

Meal weight (grams, in raw form) was equivalent to full portion size

^a^ Iron contribution of non-iron fortified rice flake, wheat and millet flour is in parentheses

### Sample description

The pilot included 387 children in grades 1–3 and 375 in grades 4–5 (Table 3). The control included 390 children in grades 1–3 and 360 in grades 4–5. The mean age was 7.6 years (1.7 SD) for the pilot and 7.7 years (1.6 SD) for the control. The mean age for children from grades 4–5 was 10.4 years (1.40 SD) and 10.5 years (1.30 SD) for pilot and control schools, respectively. Nepali was the main language spoken at home for both groups and all grades. Most children (90%) were living within an extended family or with their parents.

**Table 3:** General characteristics of schoolchildren and availability of school meal (grades 1–5)

	Grades 1–3			Grades 4–5		
	Pilot (*n* = 387)	Control (*n* = 390)	*p*-value	Pilot (*n* = 375)	Control (*n* = 360)	*p*-value
Age (years)	7.6	7.7	0.759	10.4	10.5	0.309
Gender (% of children)						
- Male	49.6	50.3	0.914	46.4	52.5	0.114
- Female	50.4	49.7		53.6	47.5	
Grade (% of children)						
- Grade 1–4	22.2	20.5	0.773	52.5	48.3	0.287
- Grade 2–5	36.7	36.2		47.5	51.7	
- Grade 3	41.1	43.3				
Home language (% of children)						
- Nepali	45.2	36.2	0.035	40.5	33.6	0.082
- Tamang	25.6	29.2		25.3	33.1	
- Tharu	21.4	22.6		25.6	23.6	
- Other	7.8	12.1		8.5	9.7	
Living arrangement (% of children)						
- Extended family	42.6	41.8	0.618	42.7	41.7	0.620
- Both parents	48.3	51.3		46.9	49.7	
- Single parent	6.5	5.4		10.4	8.6	
- Other	2.6	1.5				
Receive school meal (% of children)						
- Yes	100.0	85.1	< 0.001	99.5	83.3	< 0.001
- No	0.0	14.9		0.5	16.7	
Frequency of school meals (% of children)						
- Never to not always	14.5	32.3	< 0.001	24.0	50.0	< 0.001
- Every school day (6 times a week)	85.5	67.7		76.0	50.0	

Unpaired two-sided t-test used for age, Fisher’s exact test used for receive school meal, and Chi-square test used for all other variables

All children in the pilot schools indicated to have received school meals, while only about 84% of the children in the regular cash-based SMP did, which indicates a 16%-point increase in school meal provision (or a 19% increase). In terms of the frequency of the school meal provision, 86% of the children in grades 1–3 and 76% of the children in grades 4–5 in the pilot schools indicated to have received a school meal every day during the last six school days. These percentages are significantly higher than for the control schools, suggesting that the intervention has increased the meal provision to schoolchildren.

### Quantitative assessment of nutritional knowledge and hygiene

Children in grades 1–3 in the pilot schools had a higher mean score on hygiene practices than children in the control schools (8.8 vs. 8.3; *p* < 0.01), were better able to name fruit and vegetables (8.9 vs. 8.5; *p* < 0.01), and had better dietary practices (6.6 vs 6.1; *p* < 0.01); however, all these effect sizes were less than 10% (Table 4). More substantial was the 25% higher knowledge score for healthier snack choices (3.5 vs. 2.8; *p* < 0.01). However, there was no significant difference in children’s knowledge of nutrition and hygiene. Children in grades 4–5 in the pilot schools had significantly better hygiene practices than children in the control schools (12.2 vs. 11.8; *p* < 0.01). However, there was no significant difference in children’s knowledge of nutrition and hygiene or in their dietary practices.

**Table 4:** Children’s knowledge, dietary practices and hygiene practices

Outcome variable	Pilot		Control		% change	*P*-value
	Mean	SD	Mean	SD		
Schoolchildren in grades 1–3:						
- Knowledge of nutrition and hygiene (0–10)	8.0	1.6	7.9	1.5	1.3	0.403
- Hygiene practice score (0–10)	8.8	1.1	8.3	1.2	6.0	< 0.001
- Ability to name fruit and vegetables (0–10)	8.9	1.7	8.5	1.6	4.7	< 0.001
- Dietary practices (0–8)	6.6	1.1	6.1	1.3	8.2	< 0.001
- Knowledge of healthier snacks (0–5)	3.5	1.3	2.8	1.3	25.0	< 0.001
Schoolchildren in grades 4–5:						
- Knowledge of nutrition and hygiene (0–10)	8.4	1.5	8.2	1.5	2.4	0.095
- Dietary practices (4–20)	14.2	2.1	14.1	1.9	0.7	0.608
- Hygiene practices (0–14)	12.2	1.5	11.8	1.6	3.4	0.001

Grades 1–3: Pilot: *n* = 387; Control: *n* = 390. Grades 4–5: Pilot: *n* = 375; Control: *n* = 360. t-test used

### Quantitative assessment of dietary quality

The number of meals consumed by children in grades 4–5 over 24 h was significantly higher for the children in the pilot schools than for the children in the control schools (4.5 vs. 4.4; *p* < 0.05) as shown in Table 5. The dietary diversity score for the midday meal was 44% higher in the pilot than for the control (2.3 vs. 1.6; *p* < 0.01). The overall diversity score for all meals was also higher for children in the pilot (3.9 vs. 3.5; *p* < 0.01).

**Table 5:** Number of meals and dietary diversity scores of school meals and all meals measured using a retrospective one-day food recall method for children in grades 4–5

Outcome variable	Pilot (*n* = 375)		Control (*n* = 370)		% change	*P*-value
	Mean	SD	Mean	SD		
Meals eaten in last 24 h (0–5)	4.5	0.6	4.4	0.6	2.3	0.038
Dietary diversity score school meal (0–7)	2.3	0.9	1.6	0.6	43.8	< 0.001
Dietary diversity score all meals (0–7)	3.9	1.0	3.5	0.9	11.4	< 0.001

t-test used

**Fig. 1:**
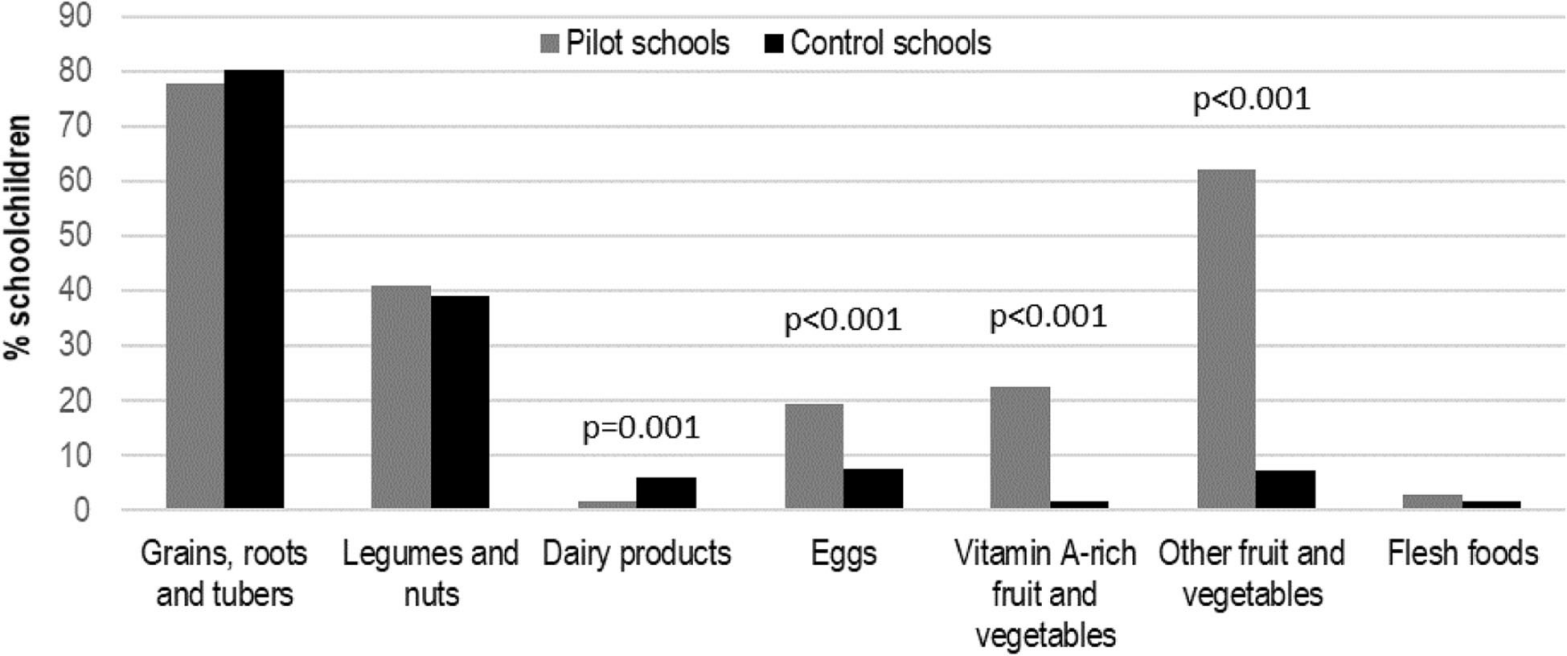
Percentage of schoolchildren in grades 4–5 from pilot and control schools consuming food from seven nutrient-dense food groups during school meals

A comparison of food groups consumed through the school meals between children in the pilot and children in the control schools shows that a much higher share of children consumed nutritious food groups including vitamin A rich fruit and vegetables (22.5% vs. 1.6%; *p* < 0.01), other types of fruit and vegetables (62.2% vs. 7.2%; *p* < 0.01), and eggs (19.5% vs. 7.6%; *p* < 0.01) as shown in Fig. 1. Yet, the share of children consuming dairy products was slightly higher in the control schools (1.6% vs. 6.0%; *p* < 0.01) while the means for the other three categories were not significantly different.

## Discussion

This pilot study tested the effect of transforming the existing cash-based SMP into a home-grown SMP with greater involvement of parents, agricultural cooperatives and local food suppliers whilst building the capacity of parents, cooks and caterers, and schoolchildren. Our results show that schools in the pilot had a significantly higher proportion of children receiving a midday meal every school day. The results also show a higher school meal quality as indicated by a higher dietary diversity score and a higher consumption of vitamin A-rich fruit and vegetables, other fruit and vegetables, and eggs. The qualitative findings confirmed that children in the pilot increased their consumption of nutritious food and reduced their consumption of junk food. Therefore, increased meal provision and increased meal quality appear as key nutritional outcomes of the pilot.

Seasonality could affect dietary diversity. We collected data in September to November when there is abundant supply from gardens and farms in Nepal. Dietary diversity can also be affected by the time of the data collection. However, both issues would affect control and pilot groups equally and both groups of schools were selected from the same locations.

The qualitative research findings point at four key drivers for these positive outcomes. The first is the use of standard meal options that are nutritionally-balanced and use locally available produce. The use of such standard menus is a low-cost intervention that could easily be scaled out to other schools. Our analysis showed that these meals provide about 27% of children’s RDA for vitamin, 20% for energy, protein, niacin and thiamin, and 10% for calcium and iron. The nutritional quality, particularly for iron and protein, could be improved. The use of affordable micronutrient-fortified food such as iron-fortified rice, high protein legumes such as soybean, and increased use of local nutrient-dense foods such as dried vegetables could be considered.

The second driver is the capacity building of parents, teachers, caterers and cooks. This helped to focus all actors on child nutrition and professionalize the school meal provision. However, this is a costly part of the intervention and these costs were also not quantified in our study. Previous studies for the United States and Lao PDR have also pointed at the importance of training and capacity building to sustain school-based health programs [27, 28].

The third driver is strengthened community ownership of the program. This was achieved in a flexible way through the use of three alternative modalities. It showed that parents associations can manage the program at smaller schools while catering may be better suited for larger schools. The pilot had a remarkable involvement of parents, cooks, agricultural cooperatives, farmers and caterers. A previous study from Mali also suggested that home-grown school feeding has the potential to lead to active participation of local stakeholders including small-scale farmers and women’s group and the school feeding program [22, 29]. Another study from Guyana revealed that parents’ and teachers’ active participation in the school feeding program was a driver to improve the quality of the program [22, 29]. Local stakeholders also played an important role in program monitoring, helping to ensure that the budget allocated to the school meal program was used in an effective and efficient manner.

The fourth and final factor is the use of local food from farmers and local retailers. The establishment of a local supply chain mechanism was important for creating community ownership. A study on school meals in Ghana also revealed that including smallholder farmers and caterers in the national school meals program improved the economy at the community level [30]. However, the steady supply of high-quality produce still is a challenge, particularly for local fruit and vegetables, which supply essential micronutrients but are highly seasonal.

Positive outcomes of the pilot were also observed in terms of better hygiene practices, a greater ability to name fruit and vegetables, and healthier food choices for the children in grades 1–3, who were exposed to the NSL component. This confirmed the findings of an earlier evaluation of the NSL component [31]. However, the effect sizes were all below 10%, except for healthier snack choices, which improved by 25%. The pilot did not improve the knowledge of nutrition and hygiene for children in grades 1–5 nor did it improve dietary practices among the children in grades 4–5. This may be attributed to the fact that dietary practices and good hygiene are already covered in the current curriculum [32]. Although useful, the NSL may not be an essential part of the intervention.

A critical aspect of the pilot program is the meal costs, as also highlighted by an earlier study [29]. We showed that the costs ranged from NPR 21.0–33.5 per meal per child with higher costs for smaller schools. Food items alone already costed NPR 13.5 while the government only provides NPR 15.0 while there is also the cost of fuel, transport, and the cook. An additional budget of at least NPR 3–5 per meal should be considered.

### Study limitations

This study is a one-time assessment and did not have baseline data to compare changes over time. The use of a randomized controlled trial design with pre- and post-intervention data would have been the preferred method of choice, but was not an option for this study. The quantitative part of the analysis assumed that the 30 schools in the control groups accurately reflect the situation in the pilot schools had the pilot not been implemented. Observable socio-economic characteristics suggest that children in the control and pilot schools were comparable, but selection bias in unobservable characteristics cannot be ruled out. Furthermore, the external validity of the study was compromised as a result of how intervention and control schools were selected. For instance, all intervention and control schools had well-functioning agricultural cooperatives and an active school management committee. The type of school meal modality adopted by schools may also affect the quality of the pilot SMP in schools. Finally, there is a chance of social-desirability bias in the self-administered questionnaire, as children could have over reported their consumption of healthy food items and underreported their consumption of less healthy food items. It is, however noted that the enumerators carefully explained that this was not a test, schoolteachers were not present in the classroom during the data collection, and children were assured about the confidentiality and anonymity of the data.

### Study strengths

A clear strength of the study is that it collected data from a large sample of 1512 children in 30 pilot and 30 control schools. The study also interviewed a large and diverse sample of stakeholders, including teachers, principals, cooks, farmers, parents and program implementers. This combination of qualitative and quantitative research methods helped to lend robustness to the findings. For instance, the qualitative data showed clear improvements in meal quality and the quantitative data confirmed this by showing improvements in the dietary diversity score and nutrient content of the school meals provided. The qualitative research also provided explanations for the observed changes, such as the use of standard menus and the importance of training cooks.

### Study recommendations

The results support the government, the WFP and other SMP stakeholders in Nepal to further develop evidence-based and cost-effective school meals programs and policies. Recommendations for further program improvement include:

- Identify ways for cost-containment, particularly looking into the wages of the cooks and hiring process; reduce the cost of the pilot SMP without reducing benefits.
- Incorporate the use of meal planning with a set menu, while allowing enough flexibility to adjust menus to seasonal variations in supplies and prices. This may require specific training for teachers and meal planners. Note that meal quality and students’ meal satisfaction were not only the result of the improved menus, but also the result of better management including the hiring a professional cook who was relatively well-paid and trained by the project.
- Improve school meal quality by increasing the micronutrient content through the use of affordable micronutrient-fortified foods such as iron-fortified rice, which is being tested in India by the WFP; increase the protein content of menu items with high protein legumes such as soybean; and increase the use of local nutrient-dense foods, including high quality, nutritious dried vegetables during some seasons.
- Improve the linkage between the SMP and local vegetable supply chains by using home garden programs, local farmers and markets.
- Encourage supply contracts with local farmers as much as possible. This would encourage local producers to supply a steady amount of nutritious foods.

## Conclusions

This assessment indicated that a change in the existing cash-based school meal program in Nepal towards a home-grown school feeding program increased the frequency of meal provision and increased meal quality in terms of dietary diversity and nutrient content. Key drivers for these improved outcomes were the use of standard meal options, capacity building of cooks and teachers, strengthened community ownership and accountability, and the linkage to the local food supply chain. Maintaining these gains would require a 20–33% increase in the current budget provision of NPR 15 per meal in addition to the costs of capacity building.

### Supplementary information

**Additional file 1.** FGD and KII guides.

**Additional file 2.** Codebook of qualitative data.

## Data Availability

The data files include (1) qualitative data (transcripts), (2) quantitative survey data, (3) pilot menu recipes, and (4) costs of the pilot program. The data files can be downloaded from 10.22001/wvc.66781 after the paper is accepted for publication. The data are anonymized.

## Appendix 1

**Table 6:** List of participants in the focus group discussions

FGD	Type of participants	Level and location of data collection	No. of participants
1.	Co-operative members and Cooks	School/community, Sindhupalchok	6
2.	Co-operative member, Cooks and focal teachers	School/community, Sindhupalchok	5
3.	Co-operative members and parents	School/community, Sindhupalchok	6
4.	School Principals	School/community, Sindhupalchok	6
5.	Principals and focal teachers	School/community, Sindhupalchok	5
6.	School Principals	School/community, Sindhupalchok	6
7.	Cooks	School/community, Sindhupalchok	5
8.	Principals and focal teachers	School/community, Sindhupalchok	6
9.	Co-operative members and farmers	School/community, Bardiya	8
10.	SMC members, parents and cooks	School/community, Bardiya	7
11.	Principals, Vice-Principals and focal teachers	School/community, Bardiya	12
12.	SMC members, cooperative member, cooks and parents	School/community, Bardiya	10

## Appendix 2

**Table 7:** List of participants in the key informant interviews

No.	Type of participant	Level and location of data collection	No. of interviews
1.	OLE Nepal: Director and content developer/trainer of nutrition sensitive literacy	National, Kathmandu	2
2.	WFP office: School Meal Program head and a team member	National, Kathmandu	2
3.	Former FFEP director	National, Kathmandu	1
4.	Department of Education: Director and deputy director	National, Kathmandu	2
5.	CDC (Deputy director)	National, Kathmandu	1
6.	Deputy District Education Officer	District, Sindhupalchok	1
7.	District Education Office: Program Officer and Focal person of SMP	District, Sindhupalchok and Bardiya	2
8.	WFP (Field staff)	District, Sindhupalchok and Bardiya	2
9.	School Principal	School/community, Bardiya	1
10.	Vice Principal	School/community, Sindhupalchok	1
11.	Cooperatives: Chairman and member	District, Sindhupalchok and Bardiya	2
12.	Cooks	School/community, Sindhupalchok	2
13.	Resource person	School/community, Sindhupalchok	3
14.	Focal teachers	Schools, Sindhupalchok and Bardiya	5
15.	Caterer	School/community, Bardiya	1

## Appendix 3

**Table 8:** Key thematic areas of FGD and KII guides

Themes	Focus of questions
Design and Implementation of SMP	A. Social protection and targeting: Universal coverage/geographic targeting/ individual targeting
	B. Program strategy focus, expected short-term and long-term impact (agriculture/ education, nutrition/health)
	C. Modalities, food baskets and nutritional norms
	D. Food procurement, transportation, storage, and preparation (several feeding supply chain models, check page 9–10, Global Feeding Sourcebook)
	E. Processing, preparation and distribution
	F. Links with local food production, small holder farmers, and local communities
Policy and legal framework	A. Policy/regulatory environment (such as international/ national treaties on school feeding and food security/nutrition etc)
	B. Source of governance (plans, guidelines, policy, law)
	C. Regulation system and its benefits
	D. Trade-offs (such as decentralized/ centralized, regulatory models)
Institutional arrangement	A. Capacity at national/subnational/ school levels to perform the designated function
	B. Coordination mechanisms with other government sectors and partners C. Resource tracking, reporting and monitoring
Funding and budgeting	A. Infrastructure investments (e.g. kitchen, dining hall)
	B. Kitchen equipment and utensils (stove, pots, pans, plates, etc.)
	C. Cost per meal portion
	D. Cost of nutrition sensitive learning program
	E. Cost of training provided
	F. Other costs
Community participation	A. Community involvement during implementation of school feeding program
	B. Key roles of the community in school feeding program
	C. Opportunity and benefits of community participation
	D. Effective participation of community participation
	E. Accountability and sustainability of community participation
Evidence of program impact	A. Any evidence generated for SMP program impact in Nepal? Reports? Publications
	B. Any plan or current activity for impact assessment?
	C. How the evidence (if generated) has/will be used?

## Abbreviations

FGD: Focus group discussion
HGSF: Home-grown school feeding
KII: Key informant interview
NPR: Nepali rupee (NPR 100 = US$ 0.89)
NSL: Nutrition-sensitive literacy
SMP: School meals program
WASH: Water, sanitation and hygiene
WFP: World Food Programme
