# Supplementary material for: Home-grown school feeding: assessment of a pilot program in Nepal
Source: BMC Public Health. 2020 Jan 8;20:28. doi: 10.1186/s12889-019-8143-9 (PMC6950908; doi:10.1186/s12889-019-8143-9)
Supplement: Supplementary file 1 — Additional file 1. FGD and KII guides. [file 12889_2019_8143_MOESM1_ESM.docx]

**FGD guide**

Participants: School management committee (SMC) members, farmers, parents, caterers, cooks etc. (5-10)

FGD time (45 mins to 1 hour 15 mins)

| **Q.N.** | **Areas of discussion** | **Additional probes (if required)** |
| --- | --- | --- |
| 1. | Can you please briefly introduce yourself, tell your role in the school meal program, and for how long you have been doing this? | - Name - Role - Since when? |
| 2. | Please describe how the school meal program is currently organized at your school? | - How do you coordinate among yourselves to implement SMP on a daily basis? - Who are the other actors (besides yourselves) involved? - What is the role of the various actors? - What support you received from WFP, MoE, and others? - How important is this support? - Who received training and how useful was this? |
| 3. | How satisfied are you with the meal quality and quantity provided to the children? | - Quality of standard menu - Advantage/ disadvantage of selecting 6-day school menu from the standard menu - How does local food availability affect the quality of selected menus? - Does cater/cook/ parents follow the menu recipes? Or frequently modified/ changed? Why? |
| 4. | What do you think of the effectiveness of the current mechanism of SMP? How is it different from the previous one? | Positive aspects?  Negative aspects?  Impact on management of SMP?  Cost effectiveness? |
| 5. | What do you think are the strengths of the current mechanism of SMP? | In terms of:  Management or smooth running of SMP?  Sustainability?  Impact on school and students?  Community level?  Personal level?  Impacts on the aspects of food security/ agriculture; education; nutrition/ health |
| 6. | What do you think are the benefits of the SMP program? | To the school?  To the students?  To yourselves (personal level)?  To the community?  Impacts on the aspects of food security/ agriculture; education; nutrition/ health |
| 7. | What do you think are the barriers of the current mechanism of SMP? | In terms of:  Management or smooth running of SMP?  Sustainability?  Impact on school and students?  Community level?  Personal level? |
| 8. | What, if any, mechanism exists to improve the SMP implementation? Mechanism to address existing challenges? | Is there a platform for you to voice the challenges that you mentioned previously? Can you tell us more about it?  Can you give us an example of how the committee addressed an existing challenge? |
| 9. | What are your suggestions for improvement of the current mechanism of SMP in your school? | How do you think we can make the program sustainable in the long run? |
| 10. | Would you like to add anything to the discussion? | |

***Thank you for your time!***

**Key Informant Interview Guide: School Level**

Participants: School principals, School teachers, Resource persons (RPs)

Approximate time: 30-45 minutes

| **Questions about the school meal program** | | |
| --- | --- | --- |
| **Q.N.** | **Areas of discussion** | **Additional probes (if required)** |
|  | Please describe how the school meal program is currently organized at your school? | Who is in charge of the overall program?  And who is in charge of:   1. Procurement, transport 2. Food storage 3. Choice of menu 4. Meal preparation 5. Hygiene and cleanliness 6. Record keeping |
|  | How is the local farming community involved in the school meal program? | What food is procured from local farmers? What food is not, or cannot, be procured from local farmers?  Are local farmers or the local community involved in any of the following activities, and if yes, how?   1. Procurement, transport 2. Food storage 3. Choice of menu 4. Meal preparation 5. Hygiene and cleanliness 6. Record keeping 7. Other activities |
|  | How are parents involved in the school meal program? | 1. Food production 2. Procurement, transport 3. Food storage 4. Choice of menu 5. Meal preparation 6. Hygiene and cleanliness 7. Record keeping 8. Other activities |
|  | What changes have been made to the school meal program over the last 12 months? | Were these changes the result of the SMM pilot?  What has improved over the previous situation?  What has gotten worse over the previous situation?  Do you expect that the improvements can be sustained in the future? |
|  | How satisfied are you with the meal quality and quantity provided to the children? | What are the challenges?  What can be done to improve meal quality?  How can the management of the school meal program at your school be improved? |
|  | Are you satisfied with the level of support provided to maintain the school meal program in its current form? | Funding provided per school meal  Investment in infrastructure  Logistical support  Technical support |
| **Questions about the nutrition-sensitive learning program** | | |
|  | Please describe me in your own words what is “nutrition sensitive learning”? | What does it aim to achieve?  How is it implemented at the school and in the classroom?  How often? |
|  | What benefits do you observe from the “nutrition sensitive learning” program? | Do the benefits vary with children’s age?  Do the benefits vary between girls and boys? |
|  | How is “nutrition sensitive learning” implemented at your school? | Who is involved in the implementation?  Are parents informed about it?  Are parents involved in it? |
|  | Are you satisfied with the contents of the “nutrition sensitive learning”? | What is good?  What is not so good?  What can be improved? |
|  | Is the “nutrition sensitive learning” supported by all stakeholders? | Is it supported by the school management?  Is it supported by the teachers?  Is it supported by the parents?  Is it supported by the children? |
|  | Would you like to add anything to the discussion? |  |

**Key Informant Interview Guide (National and District level)**

Participants: WFP, MoE, DoE, TWC members, DEO

Approximate time: 1 to 1 and half hour

| **Q.N.** | **Areas of discussion** | **Additional probes (if required)** |
| --- | --- | --- |
|  | **Design and Implementation of SMP**   1. Can you tell us about the SMP regarding the aspects of A-F 2. What are the challenges?      1. How are the modified SMMs +NSL different from the current SMP regarding the aspects of A-F 2. What are the barriers of implementing SMM+NSL | The answers should cover the following aspects:   1. Social protection and targeting: Universal coverage/geographic targeting/ individual targeting 2. Program strategy focus, expected short-term and long-term impact (agriculture/ education, nutrition/health) 3. Modalities, food baskets and nutritional norms 4. Food procurement, transportation, storage, and preparation (several feeding supply chain models, check page 9-10, Global Feeding Sourcebook) 5. Processing, preparation and distribution 6. Links with local food production, small holder farmers, and local communities |
|  | **Policy and legal framework**   1. Can you describe how the SMP fits with the existing international, national policies? 2. What is the source of governance? 3. What is the current regulation system? 4. Is there trade-off? 5. Would the modified SMM+NSL have impacts on current/future policy framework? | The answers should at least cover the aspects:   1. Policy/regulatory environment (such as international/ national treaties on school feeding and food security/nutrition etc) 2. Source of governance (plans, guidelines, policy, law) 3. Regulation system and its benefits 4. Trade-offs (such as decentralized/ centralized, regulatory models) |
|  | **Institutional arrangement**   1. How do you think the capacity at national/subnational/ school levels to perform the designated function 2. What are the coordination mechanism with other government sectors and partners 3. What are the current resource tracking, reporting and monitoring mechanism of SMP 4. Does the modified SMM+NSL have impacts on current institutional arrangement? | 1. Capacity at national/subnational/ school levels to perform the designated function 2. Coordination mechanisms with other government sectors and partners 3. Resource tracking, reporting and monitoring |
|  | **Funding and budgeting**  Need to collect secondary data on program costs for each pilot school: | Cover aspects:   1. Infrastructure investments (e.g. kitchen, dining hall) 2. Kitchen equipment and utensils (stove, pots, pans, plates, etc.) 3. Cost per meal portion 4. Cost of nutrition sensitive learning program 5. Cost of training provided 6. Other costs |
|  | **Community participation**   1. Is there community involvement during implementation of school feeding program? Who/what are they? 2. What are the key roles of the community in school feeding program 3. Does the modify SMM+NSL have impacts on community participation? How? | Questions/ answers cover the aspects   1. Community involvement during implementation of school feeding program 2. Key roles of the community in school feeding program 3. Opportunity and benefits of community participation 4. Effective participation of community participation 5. Accountability and sustainability of community participation |
|  | **Evidence of program impact**   1. Is there any evidence generated for SMP program impact in Nepal? Reports? Publications 2. Any plan or current activity for impact assessment? 3. What and how will you use the evidence (if generated) for? |  |
|  | **Do you have anything to add?** |  |

***Thank you for your time ☺***
