# Supplementary material for: Home-grown school feeding: assessment of a pilot program in Nepal
Source: BMC Public Health. 2020 Jan 8;20:28. doi: 10.1186/s12889-019-8143-9 (PMC6950908; doi:10.1186/s12889-019-8143-9)
Supplement: Supplementary file 2 — Additional file 2. Codebook of qualitative data. [file 12889_2019_8143_MOESM2_ESM.docx]

Codebook -WFP Pilot SMP

Nodes

| Name | Description |
| --- | --- |
| Benefits of the program | Parent node |
| Benefits to children | Improved nutrition, enhanced learning, child attendance and enrolment Knowledge as a result of NSL program |
| Benefits to parents | Load off of parents, opportunity for parents and community members to engage in SMP |
| Benefits to schools | Schools can focus on teaching, child attendance and enrolment, capacity enhancement as a result of trainings, etc. |
| Opportunity or benefits of community participation | The opportunity/ benefits that the inherent focus of SMP on community participant brings to the local community |
| Improved local economy | Improved economy due to local products having a reliable market, cooperatives/ caterers employed, etc. |
| Community Participation | Parent node- one of the 5 units of analysis |
| Accountability and Sustainability of community participation | Mechanism in ensuring accountability of local levels/ community, which can increase confidence and develop trust of local people affecting the sustainability of the program. Is participation from different community bodies sustainable? How can this level of participation be sustainable? |
| Com or Local involvement in pilot development | Were community people involved in the WFP pilot development? How? For example, discussions were held with local farmers about food availability based on which the menu was developed. |
| Com or Local involvement in pilot implementation |  |
| Caterers | Involvement of caterers in pilot, i.e. an independent entity who prepare food under SMP |
| Cooperatives | Involvement of cooperatives in implementation |
| Farmers | Involvement of farmers, agriculture committees in pilot, for example by supplying crops for food preparation. |
| Local businesses | Involvement of local businesses in the implementation such as shops from where schools buy everyday items such as salt or fuel. |
| Other local committees | Involvement on any local committees such as nutrition committees, child clubs, mother’s groups, and health posts, etc. in implementation of the pilot EXCEPT schools, cooperatives, caterers, parents, farmers and local businesses |
| Parents | Involvement of parents in implementation such as in committees, arrangement of fuel, as cooks, etc. |
| Cooks | Cooks responsible for cooking school meal in the school, could be parents as well |
| Schools | Involvement and responsibilities of schools in implementation Double code: Role of school in implementation |
| Opportunity and benefits of community participation | Current/ future benefits of community participation Opportunities of current participation Opportunities for participation in future |
| Comparison between past or other programs and WFP implemented SMP | Any comparison (positive/ negative) or observation in the current pilot and the previous SMP program implemented by JICA or other organization. |
| Design and Implementation | Parent node-One of the five areas of analysis |
| Background of the WFP pilot | How/ what influenced the WFP pilot, how did the concept emerge, who were involved in designing the pilot at the central level NOT district/ local level, what procedures were followed, e.g. meetings with national level stakeholders to discuss the various modalities under piloting donor requirements |
| Catering model | Information specific to catering model such as implementation of this model, procurement, storage, preparation and other activities under this model, etc. |
| Challenges encountered during piloting |  |
| Attitude or perception of local level bodies towards the pilot | Negative attitude/ perception of local bodies towards pilot For example, schools dissatisfied with extensive monitoring/ documentation and because under the pilot they have to share responsibility with cooperatives |
| Challenges in establishing local supply chain | Challenges such as small scale farmers not able to supply all required crop products in the required amount |
| Dissatisfaction with cooperatives, with WFP program | For example: local businesses upset because cooperatives from another VDC was contracted in Sindhupalchowk and resources came from another VDC Schools dissatisfied with cooperatives. Dissatisfaction on some issues of WFP program |
| Geographical barriers | Barriers/ Challenges due to geography, terrain |
| Lack of awareness | Lack of awareness among the parents and in the community about the program and its benefits, caste issues etc |
| Recording or reporting challenges | Improper/ inaccurate recording or reporting or any other challenges related to recording or reporting in the pilot |
| Resource related challenges | Any challenge related to resources, primarily money/ equipment/ human resources during the pilot |
| Stakeholders not fulfilling their roles | Stakeholders (individual or organization), especially local level stakeholders such as parents, caterers, schools, etc. not performing the expected roles. Stakeholders exhibiting fraudulent behaviour. |
| Distribution to students | Mechanism followed from the point of food preparation to food distribution to students, for example, attendance record sent to kitchen, children form queue, older children get more portion, etc. |
| District or Community or Local involvement in pilot development | Who were involved at the district and community level during development of the pilot and how, for example maybe be schools provided some suggestions or attended the discussion while the pilot was being designed. |
| Efforts to curb challenges | What efforts have been made during the pilot by different stakeholders (central, district, local) to curb challenges and improve the situation |
| Food basket and nutritional norms | Range of feeding modalities: hot meal to a snack to take-home rations. Food basket: diversified food including freshly produced or local products for school meal Nutritional norms: Nutritionally balanced menus specifying the food types and their calorific value covering daily nutritional needs of students |
| Food processing and preparation in the pilot | Mechanism of food processing and preparation in the pilot, prepares food, role of local bodies such as schools, nutrition committees. Quality assurance during meal preparation. For example, school checks the quality and work of cook and gives feedback Quantity and quality of food, what do stakeholders/ children think about the quantity and quality of food CODE HERE IF NOT CLEAR WHICH SMM |
| Food procurement, transportation and storage process in the pilot | What is the process of food procurement, transportation and storage in the pilot |
| Role of local stakeholders in procurement and transportation | Role of local stakeholders such as cooperatives/ schools/ parents/ farmers/ local shopkeepers |
| Kitchen gardening pilot and SMP | Initiative, progress, process or any other information associated with kitchen gardening in the pilot or the general SMP program |
| Menu in pilot |  |
| Children's satisfaction with meal on menu | How are the children liking/ not liking the food on the menu |
| Effect of local food availability on menu | What is the effect of local food availability, seasonal variations on food menu? Any difficulty in sticking to the menu as a result of local food availability. |
| Feedback or perception on menu | How do stakeholders (central/ district/local) feel about the menu currently being used in the pilot NOT feedback of children |
| Food preparation according to menu (Yes or No) | Are the meals being prepared according to the menu or not? Reasons for sticking/ not sticking to the menu. |
| Process of menu development | How was the menu developed, consideration of nutritional content, local food availability, etc. |
| Suggestions on menu | Any suggestions for modification or improvement in menu(DOUBLE CODE) under broad heading suggestions-suggestions to change menu |
| Monitoring, recording and reporting mechanism | Mechanism of monitoring from higher levels, frequency, content of monitoring, impact of monitoring Mechanism of reporting and reporting from local to district to central levelANY SPECIFIC INFORMATION ON THE THREE SMMS TO ALSO BE CODED UNDER THE THREE SMMS CODE |
| NSL Pilot | Background and concept of NSL piloting, its development, who were involved in its development and implementation, components of NSL piloting, implementation mechanism, target group, Link between NSL and SMM Pilot |
| Benefits of NSL pilot | What are the benefits or impacts of the program |
| Challenges of NSL pilot | All the challenges related to NSL pilot |
| Content of NSL | What is there in the curriculum, how satisfied were the implementers with the content, what are the good aspects and what needs to be improved in the content, resource used in NSL (NOT overall suggestions) |
| Implementation of NSL pilot | How NSL pilot is being implemented, who are involved, target group etc. |
| Objectives | What are the objectives of NSL pilot? |
| Suggestions for NSL pilot | Overall suggestions (NOT on the content) regarding NSL pilot implementation |
| Support from different stakeholders in NSL implementation | Support from School management committee, teachers, parents and students. |
| Objectives of the pilot | The short-term and long-term objectives of the pilot |
| Parent association model | Information specific to parent association model such as implementation of this model, procurement, storage, preparation and other activities under this model, etc. |
| School management model | Information specific to school management model such as implementation of this model, procurement, storage, preparation and other activities under this model, etc. |
| School Meal modalities | Information specific to three different models such as implementation of these models, procurement, storage, preparation and other activities under these model, comparison between these models etc. |
| Selection of study sites for piloting | How the pilot sites were selected, the criteria. |
| SMP-General |  |
| Background or History of SMP program | How/ what influenced the current design and implementation mechanism, How did the current SMP program evolve/ develop/ transpire Why is SMP important? |
| Cash based SMP modality | Anything to do with the cash-based SMP modality, e.g. Implementation mechanism, procurement, transportation, reporting related to cash based SMP modality |
| Challenges of SMP | Any challenge of the general SMP program |
| Food based SMP modality | Anything to do with the cash-based SMP modality, e.g. Implementation mechanism, procurement, transportation, reporting related to cash based SMP modality |
| Goals or objectives of SMP | What are the long-term and short-term goals/objectives of the general SMP? |
| SMP as Social protection measure | Link of the concept of school meals to social protection |
| Strengths or Opportunities of SMP | Strengths or opportunities For example, positive attitude/ behavior of stakeholders. For example, willingness of stakeholders at different levels to support SMP |
| Suggestions | Suggestions to overcome the challenges during implementation of SMP |
| Targeting mechanism | What approach has been followed for targeting: Universal/ Individual/ Geographic Who are the target groups? |
| Strategic focus or multi-sectorality | What is the strategic focus of the SMP/ Pilot, involving multiple sectors like agriculture, education, health and nutrition? |
| Strengths or Positive aspects of the pilot |  |
| Equipment managed | Utensils, improved stove, storage containers, and other infrastructure managed during pilot |
| Food quality or hygiene | Strengths related to food quality in terms of hygiene, taste, quality of ingredients used, cleanliness, nutrition content, etc. |
| Kitchen garden | Concept of kitchen garden in schools |
| Menu consistency | Strengths of menu such as nutritional content, diversity and local availability considered DOUBE CODE with ‘Feedback/ perception on menu’ |
| Money for cooks | In the piloting there is money allocated for cooks |
| Training | Training provided as part of the pilot Example, participants appreciating training provided on book-keeping and meal preparation |
| Suggestions for improvement from WFP pilot |  |
| Cooperative management | Suggestions regarding effective management with cooperatives |
| Suggestions for central level | Suggestions that apply to central level stakeholders or that can be employed in district level |
| Suggestions for district level | Suggestions that apply to district level stakeholders or that can be employed in district level |
| Suggestions for local level | Suggestions that apply to local level stakeholders or that can be employed in local level |
| Suggestions to change menu | Suggestions regarding modifications in menu DOUBLE CODE with ‘Suggestions to change menu’ under broad code ‘Menu in pilot’ |
| Training suggestions | Suggestions regarding training need, training content, participants, duration, frequency |
| Target group or beneficiaries of WFP pilot or SMP | Example children, indirect beneficiaries such as farmers, schools, parents, etc. |
| Evidence of program impact | Additional Parent Code |
| Any plan or program for impact assessment | Any plan/ program by WFP/ GoN/ other body for impact assessment |
| Impact of evidence generation | What can be the impact of the evidence generated from the pilot/ other evidence regarding SMP on program improvement, sustainability, efficiency, strategy/ policy formation etc? |
| Past evidence of SMP in Nepal | What does the literature say about the implementation mechanism, effectiveness, impact, etc. of SMP? |
| Funding and Budgeting | Parent node- one of the 5 units of analysis |
| Budget allocated and expenditure for school feeding | How much/ what percentage of budget is allocated for school feeding from government/ WFP? What amount is being spent on school feeding yearly, any comments on this amount relative to educational expenditure Food cost: What is the total cost incurred on different food items for school meal? |
| Budget disbursement mechanism, Top to bottom | What is the mechanism of budget disbursement from the funders to central level stakeholders to district level stakeholders to the implementers at local level What is the mechanism of budget disbursement for the three piloting modalities? Schools get budget from DEO & then they pay cooperatives in all 3 models. Mechanism of budget related reporting: How do schools/ cooperatives and other local bodies report to district and higher levels on budget spending, process of approval, process of reimbursement, etc |
| Funders | Who is/ are funding the various aspects of the pilot? For example, government is funding the school meals, but WFP is funding the cooks. Funding translates to money but also equipment such as utensils. |
| Sufficiency of funding | Is the funding allocated for different aspects of the WFP pilot sufficient? How? Why? |
| Impact of earthquake on SMP or pilot | What was/is the impact of earthquake on SMP or pilot? |
| Impact of SMP or pilot | Parent node |
| Impact on community | Perceived/ actual impact of the SMP/ pilot on the community |
| Impact on teacher or parents or personal level or cooks | Perceived/ actual impact |
| Institutional Arrangement | Parent node-one of the 5 units of analysis |
| Actors and their roles |  |
| Central level stakeholders and their roles | Who are the central level stakeholders (eg. MoE/ WFP) and what are their expected roles in SMP/ pilot? |
| District level stakeholders and their roles | Who are the district level stakeholders (DoE, District WFP staff, others) and what are their expected roles in SMP/ pilot? |
| Local level stakeholders and their roles |  |
| Caterers | Expected role of caterers in implementation of Pilot/SMP implementation |
| Cooperatives | Expected role of cooperatives in implementation of Pilot/ SMP implementation |
| Farmers | Expected role of farmers, agriculture committees in implementation of Pilot/SMP implementation |
| Other local level stakeholders | Who are the local level stakeholders and what are the roles expected from then for the pilot/SMP implementation? |
| Parents | Expected role of parents in implementation of Pilot/ SMP |
| Schools | Expected role of schools in implementation of Pilot/SMP |
| Capacity of stakeholders to perform designated function | Existing capacity of national, district and local level stakeholders to perform designated function, e.g. Lack of proper infrastructure, inadequate staff, inadequate space, untrained staff, etc. Any gap in capacity (need for training) |
| Coordination mechanism |  |
| Horizontal i.e. between stakeholders at each level | How do stakeholders coordinate with each other at the same level? Eg. At the local level, what is the mechanism of coordination between schools, farmers, parents, cooperatives, etc. |
| Vertical i.e. across different levels | How do central, district and local level stakeholders coordinate with one another? Medium- e.g. supervision and monitoring visits, telephone conversation, review meetings, etc. How often? Is there a clear structure/ guideline for coordination? |
| Lead agency and their functions | Who is the lead agency for the school meals? |
| Resource tracking, reporting and monitoring | What is the structure of resource tracking, reporting and monitoring from central to district to local levels and vice versa. |
| Support from different organizations | Any organizations are playing a supportive role in the SMP program/ pilot, what is their role E.g. advocacy Organizations at central/ district/ local level |
| Training and capacity building | Anything related to training such as training frequency, duration, location, who the trainers are, who are the recipients, what are the contents of the training, etc. Any other activity contributing to capacity building of stakeholders such as conferences, sharing meetings if any. |
| Link to new federal structure | Any link of the SMP program/ pilot to the new federal structure |
| Participant Information | Participant's introduction and their role in SMP |
| Policy and Legal Frameworks | Parent node- one of the 5 units of analysis |
| Centralized or decentralized approach to SMP implementation | What approach are we using with SMP in Nepal, centralized or decentralized? |
| Existing executive or legal bodies | What are the sources of governance in relation to the SMP/ WFP pilot, what are the bodies that are involved in formulating in SMP related policies (direct/ indirect), any government/ non-government bodies that play an active role in advocating for polices and strategies related to SMP. Government/ governance bodies implementing/ playing a role in implementing SMP/ pilot |
| Existing policies or strategy or guideline | Current policies/ strategies/ guideline on SMP, for example Equity Strategy |
| Linkage of national strategy or guideline with international guideline | How does our national policy/ strategy/ guideline link with its international counterparts |
| SMP i.e. linkage to 3 rights (food, education and health) | How SMP can be linked to or addresses3 rights i.e. right for food, right for education and right for health |
| Ragbag or Miscellaneous | Codes that do not fit under any of the existing codes but seem important. |
| Sustainability of the program | Parent node |
| Challenges to sustainability | What are the current/ potential threats to sustainability of the SMP |
| Evidence generation and potential impact on sustainability | Evidence from the pilot or other evident and its impact on the sustainability of SMP |
| Suggestions for sustainability | How can the SMP be made sustainable? How can aspects of the pilot be made sustainable? |
| Sustainability of NSL | Is NSL sustainable? Why? Why not? Support/ advocacy from different groups. |
| Sustainability of SMP | Is SMP sustainable? Why? Why not? Support/ advocacy from different groups. |
